# Do traditional masculine norms predict emotion regulation? A cross-cultural quantitative study of urban Indian men

**DOI:** 10.3389/fsoc.2026.1803155

**Published:** 2026-06-04

**Authors:** F. Francis Kulandai Raj, V. Vijayalakshmi

**Affiliations:** School of Social Sciences and Languages (SSL), VIT University Chennai, Chennai, India

**Keywords:** cross-cultural psychology, gender norms, India, masculinity, men’s emotions, sociology of emotion, urban men

## Abstract

**Introduction:**

Western studies on psychology and emotion have elucidated that higher practice of traditional masculine norms (TMN) is linked to limited ways of handling emotions, especially holding back feelings among men. However, the research gap indicates that there is insufficient empirical research conducted on cultural and population-based factors related to this issue.

**Methodology:**

The study utilized a quantitative, cross-sectional correlational design. Snowball sampling was employed for data collection through an online survey of 151 urban, educated men age 18–39 years (*M* = 26.70, SD = 4.6) from Chennai, India. The respondents for the study received a bilingual (English and Tamil) tool that consists of the Emotion Regulation Questionnaire (ERQ) and a self-structured Traditional Masculine Norms (TMN) scale adapted from the MRNI-R and CMI-46. Data were analyzed using EFA, CFA, Pearson correlations, multiple regression, chi-square tests, and ANOVA.

**Results:**

Factor analysis validated a two-factor TMN structure (Rigid Masculine Role and Homophobia/Transphobia) with excellent fit (CFI = 0.991, RMSEA = 0.041). The key finding contradicts existing theories that suggest a close relationship between gender ideologies and emotion expression, as TMN and emotional regulation strategies (CR/ES) operate independently, rejecting H2. Demographic variables did not predict emotion regulation (H3a rejected), but religious affiliation significantly predicted lower TMN conformity, partially supporting H3b. A majority of respondents (65.6%) exhibited a shift in gender attitudes, characterized by low TMN attitudes.

**Discussion:**

The null findings contradict the universal applicability of the Gender Role Strain Paradigm, suggesting that in this urban Indian context, masculine ideology and emotion regulation functions independently. Emotion regulation strategies may function as context-specific “emotion work” governed by situational feeling rules rather than as an expression of global masculine identity.

## 1 Introduction

Men and masculinity studies in connection with gender norms and emotion have received increased attention in the social sciences recently. For decades, Western studies in psychology have adopted a theoretical narrative that conformity to traditional masculine norms, which consist of a certain set of traits like dominance, stoicism, self-reliance, and emotional restraint, impacts men’s emotional aspects. The narrative was formally articulated within the Gender Role Strain Paradigm (Pleck, 1995), which argues that internalization of this norm leads to restrictive emotionality, where suppression of certain vulnerable emotions becomes a standard eligibility of manliness. Several studies (Cleary, 2012; Courtenay, 2000) from Europe and North America show that following traditional masculine beliefs is linked to hiding feelings instead of contemplating them differently, which harms men’s mental health and close relationships. The health risks are higher among men who adhere to traditional masculine norms as compared to men who do not adhere to the same. The suppression of emotions, particularly distressing ones, increases the risk of suicidal ideation and behavior in men.

This established notion overlooked the fundamental social construction of gender and emotion. From a sociological lens, gender is not personal. It is a social institution, which creates a set of roles and responsibilities that organize social life (Lorber, 1994). Likewise, emotions are not private; they are deeply interconnected with social phenomena. Sociologists of emotion argue that what an individual feels, how she/he/they interpret those feelings, and the way they express them are culturally specific and learned through socialization that dictates an individual express certain emotions in a given situation (Hochschild, 1979; Stets, 2010; Thoits, 1989). In other words, the emotions we express are governed by “feeling rules” (Berke et al., 2018; Hochschild, 1983; Özkaya, 2025; Stets, 2012; Thoits, 1989).

Hence, the connection between masculine norms and emotional regulation strategies must be examined and understood from the cultural lens outside the Western context. It is integral to conduct a theoretical interrogation in culturally diverse countries like India, where socio-cultural aspects are complex and shaped by ancient philosophical systems, the caste system, diverse religious moralities, colonialism, and post-liberalization modernity. Considering these contexts, masculinity is constructed as a dynamic process. Simultaneously, urban education, feminist perspectives, media, and digital technology reshape it. This development provided a space for men to express the traditional masculine behaviors, selectively retained or reinterpreted through a modern lens, which scholars term “hybrid masculinities” (Bridges and Pascoe, 2014).

The study aims to empirically test the Western-derived concept of gender role strain in an urban Indian space, such as the city of Chennai (in South India), which is a non-Western nation, through the social constructionist and cross-cultural sociological lens. The research aims to answer important questions, such as (i) Does the theoretically argued and empirically proven connection between TMN (traditional masculine norms) and expressive suppression yield consistent results in Indian cultural settings? (ii) Do demographic factors, including religion, marital status, income, and age, influence masculine norms among men and shape psychological constructs in India?

## 2 Theoretical frameworks

### 2.1 Gender role strain paradigm

The Gender Role Strain Paradigm (GRSP), by Pleck (1995), is one of the significant theories relevant to the psychology of men and masculinity. GRSP argues that gender norms are socially constructed, and whoever adheres to or violates those norms can face psychological consequences. Essentially, it critiques traditional masculinity ideologies for creating norms that encourage men to suppress specific emotions, exhibit aggressive behavior, and avoid seeking help (Levant and Powell, 2017; Levant and Richmond, 2016). There are three types of strains: Firstly, discrepancy strain, which describes that men undergo stress if they fail to meet or conform to masculine ideals. Secondly, dysfunctional strain, under which men face negative consequences for conforming to certain masculine norms, such as aggression and emotional suppression. Thirdly, trauma strain applies to a specific group of men who have experienced harsh socialization, including men of color, LGBTQ+ individuals, and survivors of child abuse (Levant and Richmond, 2016). Masculinity ideologies are all about beliefs, which contain a set of roles and behaviors that are appropriate for men. However, these ideologies differ by class, culture, and political and historical context but often encourage dominance, emotional restraint, and self-reliance, which can lead to negative mental health outcomes for men who feel pressured to conform to these norms. The theory posits that reconfiguring masculinity can mitigate stress and improve well-being.

### 2.2 Gross’s process model of emotion regulation

The Emotion Regulation Questionnaire (ERQ) is associated with Gross’s process model of emotion regulation (Gross, 1998). This model measures and distinguishes between two primary strategies: *cognitive reappraisal*, a strategy that involves an antecedent focus, reducing or preventing stress before the impact of full emotional responses occurs by changing one’s interpretation of a situation, which is also usually linked with positive emotional experiences, good interpersonal functioning, and better psychological well-being. *Expressive suppression* is a strategy that involves deliberately and intentionally hiding the outward emotional behaviors that are linked with low social support and well-being. This model has been sufficiently validated across cultural contexts (Butler et al., 2003; Cabello et al., 2013; Chen et al., 2025; Gross and John, 2003; Preece et al., 2020; Tamir et al., 2024). These two strategies are having distinct psychological and physiological connections. By applying this theory, the study is trying to test the relationship between traditional masculine norms and the suppression of emotional expressiveness in urban Indian men.

### 2.3 Integrating the two perspectives

**Figure 1:**
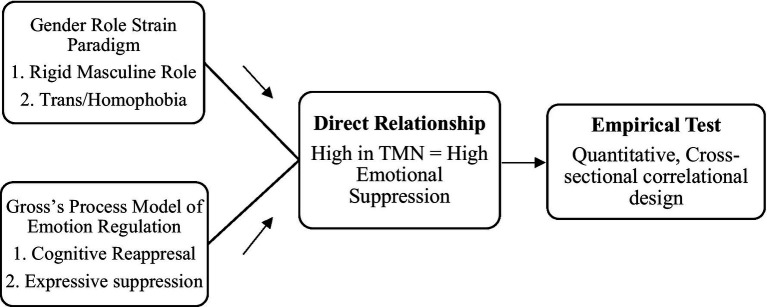
Integrated theoretical framework.

The Gender Role Strain Paradigm (Pleck, 1995) provides a foundation for hypothesizing a direct correlation between masculine norms and emotion regulation strategies. To be specific, it expects that men who conform to TMN will have a high possibility to exercise expressive suppression over cognitive reappraisal. Through these theories, the study intends to understand how the urban, educated Indian population may moderate the relationship between masculine ideology and emotional processes (Figure 1).

## 3 Literature review

### 3.1 Masculinity and emotion

Extensive Western research has examined the relationship between traditional TMN and ER strategies. Those studies consistently report that conformity to TMN is strongly linked to a greater use of expressive suppression, and lesser use of cognitive reappraisal (Levant and Wimer, 2014; Wong et al., 2017). This pattern is called “restrictive emotionality” (Levant and Richmond, 2016; Pleck, 1995), and has been influencing negative mental health outcomes such as anxiety, alexithymia, and depression and restricting self to seek psychological support (Hammond, 2012; McKenzie et al., 2018; Rice et al., 2011). Some longitudinal and experimental studies indicate that men who strongly adhere to traditional masculine ideals of stoicism and self-reliance exhibit sensitive physiological arousal during emotional tasks and have lower emotional awareness (Berke et al., 2018; Cohn et al., 2010).

Research also suggest that these patterns are learned through the gender socialization process from family members, friends, and media, starting in childhood and reinforced throughout adolescence (Addis and Mahalik, 2003; O’Neil, 2008). The practice of restrictive emotionality not only impacts individual well-being, but it also extends to interpersonal functioning; men who suppress emotions have lower relationship bonding and higher expression of aggression (Lynch et al., 2018; Tager et al., 2010). Additionally, restrictive emotionality has been an influencing factor in higher suicide rates among males compared to females in many Western countries (Cleary, 2012; River and Flood, 2021). However, this evidence comes from American and Eurocentric samples, raising the question of whether the same can be generalized to non-Western countries like India, where cultural context may play a major role in the functioning of different feeling rules and relational norms in shaping emotional expression differently.

### 3.2 Understanding Indian masculinities

Ethnicity, caste, class, religion, disabilities, and sexual orientation significantly influence the lives of men in various parts of the country. Therefore, it is extremely challenging to theorize Indian masculinity and conduct a comprehensive investigation into the vast and complex realities of Indian men (Dasgupta and Gokulsing, 2013; Mahalingam and Balan, 2008). Anthropological research on gender in India has identified notions of caste purity, caste superiority, and chastity with other castes as culturally accepted norms of “masculinity.” A predominantly pan-Indian cultural inclination has emphasized the importance of defining “masculinity” as the capacity to protect family and kin while maintaining the “purity of women and caste identity” (Osella and Osella, 2006; Yim and Mahalingam, 2006). However, an increase in urbanization and educational expansion have fostered “hybrid masculinities” where men selectively retain or reinterpret the traditional expectations through a modern lens (Bridges and Pascoe, 2014; Srivastava, 2015a). Studies indicates that urban, educated Indian men may hold more progressive gender attitudes than previous generations (Derne, 1995; Desai and Kulkarni, 2008). So far, the specific relationship between TMN and emotion regulation strategies has not been quantitatively examined in the Indian context.

### 3.3 Hypotheses

The following hypotheses were formulated based on the theoretical concepts of gender role strain in psychology and psycho-socio-cultural perspectives to be empirically tested:

*H1*: Factor structure hypothesis.

The Traditional Masculine Norms (TMN) scale in urban India, will have multiple dimensional factor structure.

*H2*: Direct relationship hypothesis (testing GRSP).

Conformity to TMN will be positively correlated with expressive suppression (H2a) and negatively correlated with the use of cognitive reappraisal (H2b).

*H3*: Institutional embeddedness hypotheses.

Demographic variables (religion, income, age, and marital status) will significantly predict emotion regulation strategies (H3a) and TMN conformity (H3b).

## 4 Method

### 4.1 Research design

The research utilized a quantitative, cross-sectional, correlational design. It employed a survey methodology retaining standardized and adapted instruments, in combination with a scale validation factor, to examine the relationships between Traditional Masculine Norms (TMN), Emotion Regulation strategies (ER), and demographic factors among a sample of urban Indian men from Chennai. Men aged 18 to 40 years who are the respondents for the study received a bilingual (English and Tamil) questionnaire to facilitate their understanding. The study obtained data from a sample of 170 Indian males from urban areas in Chennai. Finally, after screening the responses, only 151 samples were considered for the study. Snowball sampling was employed for data collection (Table 1).

**Table 1:** Demographic profile of the respondents.

Variable	Sample (*N* = 151)	
	Frequency	%
Age		
M	26.70	
SD	4.6	
Range	18–39	
Gender		
Male	151	
Religion/non-religious		
Hindu	100	66.2
Islam	3	2.0
Christian	32	21.2
Atheist	15	9.9
Others	1	0.7
Relationship status		
Married	40	26.5
In a relationship	13	8.6
Single	98	64.9
Annual income (Rs)		
Less than 100,000	49	32.5
100,000–200,000	23	15.2
200,000 – 5,00,000	44	29.1
More than 5,00,000	35	23.2
Community		
Scheduled tribe (ST)^1^	2	1.3
Scheduled class (SC)^2^	19	12.6
Other backward Class (OBC)^3^	66	43.7
Forward class^4^	17	11.3
Prefer not to mention	47	31.1

^1^Article 342 of the Indian Constitution: STs are indigenous tribal communities facing geographical and social isolation (Government of India, 2019a, sec. Article 342). ^2^Article 341 of the Indian Constitution: SCs are historically marginalized castes (formerly “Untouchables”) (Government of India, 2019b, sec. Article 341). ^3^OBCs are socially and educationally disadvantaged castes listed by the Mandal Commission 1990. (Mandal Commission Report, National Commission for Backward Classes, Govt. of India) (National Commission for Backward Classes, 2021). ^4^Forward Classes *or General Category, Historically privileged and ineligible for caste-based quotas (Supreme Court of India, 1992).

### 4.2 Data collection process

The self-structured questionnaire on masculine norms has been approved by gender study experts at the university with which the researcher is affiliated. The researcher distributed the link to the Google Form questionnaire via various online platforms, including WhatsApp, email, and Facebook. The questionnaires were completely anonymous in nature and did not ask for any personal information about the respondent. The online distribution of the questionnaires poses a challenge in calculating the response rate.

### 4.3 Ethical considerations

The study was conducted in accordance with established ethical guidelines. All procedures were reviewed and approved by the Institutional Ethics Committee at Vellore Institute of Technology, Chennai (Ref. VIT/IECH/CC/2024/60). The study maintained respondents’ anonymity, with no personally identifiable information collected. Data were stored securely on password-protected systems accessible only to the research team. The questionnaire was designed to minimize psychological risk by avoiding sensitive trauma-related content. Informed consent was obtained from all respondents digitally before survey commencement. Consent included acknowledgment of their right to withdraw at any point without consequence. Only respondents providing affirmative consent were able to proceed to the study instruments.

### 4.4 Research instrument

#### 4.4.1 Standardized scale: emotion regulation questionnaire

Respondents answered each question on the Emotion Regulation Questionnaire (ERQ) (Gross, 1998), which has 10 items and a “7-point Likert scale” that consists of “1 (strongly disagree) to 7 (strongly agree)”. The ERQ measures the frequency of “cognitive reappraisal (CR) (1, 3, 5, 7, 8, 10) and expressive suppression (ES) (2, 4, 6, 9)” of the respondent. Cognitive reappraisal scores “range from 6 to 42”; the higher the score, the more it indicates that the respondent has a tendency to use cognitive reappraisal in their emotional life. Expressive suppression scores “range from 4 to 28”; the higher the score, the more it indicates the respondent’s capability to implement “expressive suppression” in their emotional life.

#### 4.4.2 Adapted scale: traditonal masculine norms scale

The researchers also constructed a self-structured questionnaire with 10 items to measure men’s traditional masculine norms. This scale consisted of a “7-point Likert scale ranging from 1 (strongly disagree) to 7 (strongly agree)”. The researchers adapted some items from the “Male Role Norms Inventory - Revised (MRNI-R)” (Levant et al., 2010) and the “Conformity to Masculine Norms Scale (CMNI-46)” (Parent and Moradi, 2009).

#### 4.4.3 Scale development and content validation

The Traditional Masculine Norms (TMN) scale was developed through multi-stage process. In the initial stage 10 items was generated based on a review of existing Western masculinity scales (Levant et al., 2010; Parent and Moradi, 2009), through literature on Indian masculinities (Bhasin, 2004; Chowdhry, 2015; Derne, 1995; Osella and Osella, 2006) and by qualitative pilot interviews with five urban Indian men aged 18–35 to identify culturally relevant masculine believes. The 10 items were reviewed by three experts from the field of gender studies, psychology and sociology. Experts have checked the items to see whether they match with relevance to traditional masculinity in the Indian context, clarity, and cultural correctness.

#### 4.4.4 Rationale for scale adaption

The rationality for self-structured scale is to have culturally relevant items. Although there are well-established Western scales that have strong psychometric properties, they were developed in Euro-American contexts, and they may not resonate with Indian samples and lead to cultural bias (e.g., items about hunting, car racing, or specific Western gender role expectations). Instead of imposing a culturally foreign instrument, the researcher adopted a hybrid approach where it retains theoretical continuity with Western frameworks while ensuring cultural relevance, with indigenously developed items based on pilot interviews with urban Indian men. This approach is possible and considered a best practice in cross-cultural psychology (Byrne, 2016; Lawshe, 1975; Matsumoto and Yoo, 2006).

### 4.5 Data analysis

The SPSS version 22 and AMOS version 21 packages were used for analysis. The analysis process consists of three phases: scale validation and psychometric evaluation, descriptive and correlational analysis, and inferential analysis.

#### 4.5.1 Phase 1: scale validation and psychometric evaluation

Initially, reliability (Cronbach’s Alpha) and item-total correlations were tested to identify poorly performing items. Exploratory Factor Analysis (EFA) was conducted on Traditional Masculine Norms (TMN) to identify their underlying factor structure using principal axis factoring with varimax rotation. To assess suitability, the Kaiser-Meyer-Olkin (KMO) measure and Bartlett’s test of sphericity have been employed for the study. Moreover, to verify the factor structures of the improved Traditional Masculine Norms (TMN) and the Emotion Regulation Questionnaire (ERQ), a Confirmatory Factor Analysis (CFA) was conducted using maximum likelihood estimation.

Model fit is tested using the *χ*^2^/df ratio, Comparative Fit Index (CFI), Tucker-Lewis Index (TLI), and Root Mean Square Error of Approximation (RMSEA). Standardized factor loadings and factor correlations were examined. For further validation, comprehensive scores were calculated for TMN and its two subscales. The respondents’ scores were then categorized from high to low, conforming to masculinity profiles using the 25th and 75th percentile cut-points on each subscale. To categorize emotion regulation profile average scores, median splits have been employed.

#### 4.5.2 Phase 2: descriptive and correlational analysis

Descriptive statistics and bivariate Pearson correlations were calculated to test the relationships between the two factors of the masculine norms scale, the emotion regulation questionnaire, and the demographic variables, which include age, marital status, and annual income.

#### 4.5.3 Phase 3: inferential analysis

Separate standard multiple regression analyses were conducted to identify the best linear combination of age, religious affiliation, marital status, and annual income for TMN and ERQ. Chi-square tests of independence were conducted to determine the connection between the categorical masculinity profiles and other categorical demographic variables such as religion and marital status. Also, a series of one-way ANOVAs were conducted to examine the differences in emotion regulation scores among groups based on marital status and income.

## 5 Results

### 5.1 Phase 1: psychometric properties of the measures

**Figure 2:**
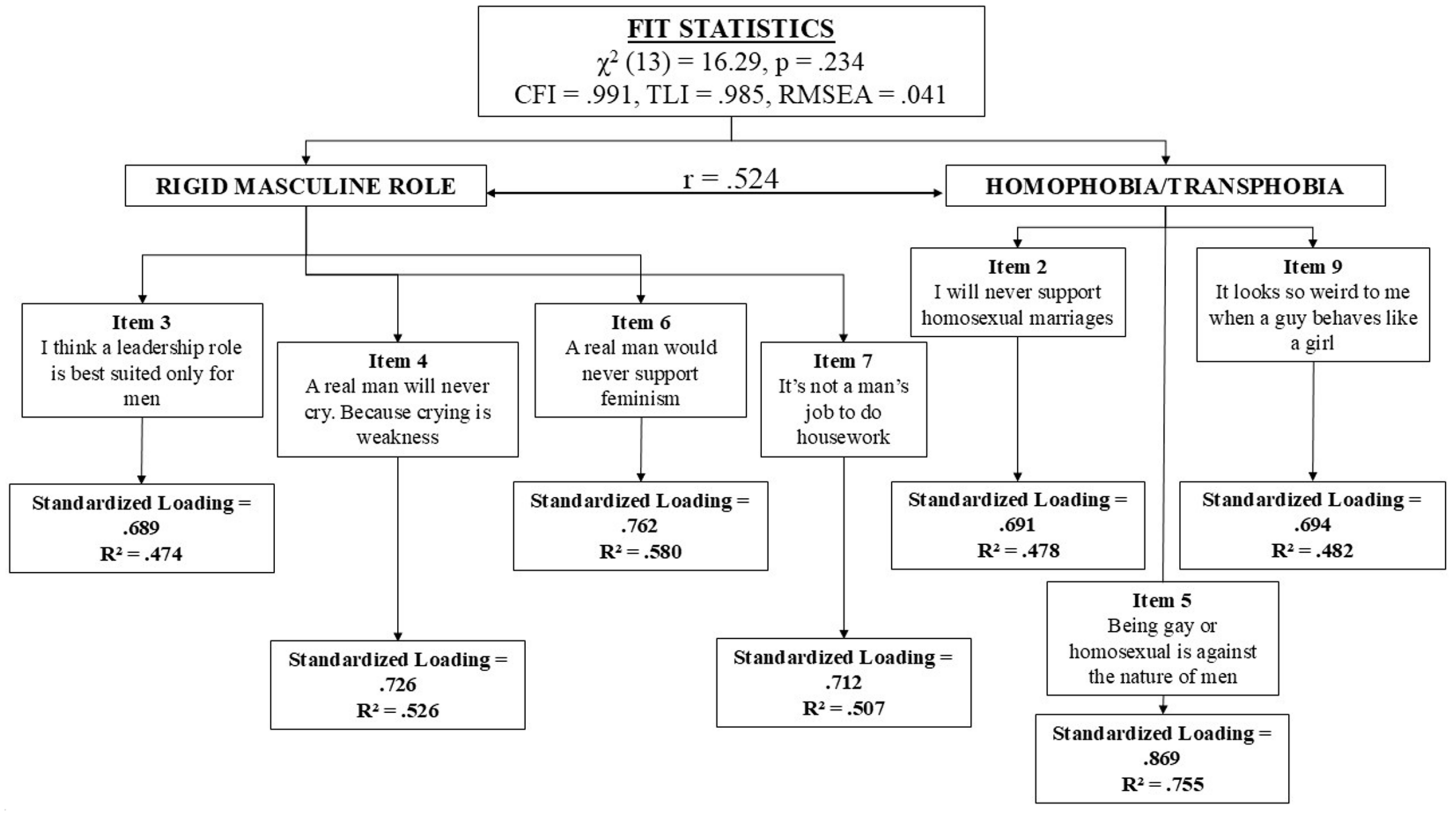
Confirmatory factor analysis results for the two-factor traditional masculine norms (TMN).

Testing of the validity of the 10-item Traditional Masculine Norms (TMN) has resulted in the removal of one poorly performing item (Item 8). The refined 9-item scale showed excellent internal consistency (Cronbach’s *α* = 0.849). EFA on the 9-item revealed a two-factor dimension (KMO = 0.845, Bartlett’s test *χ*^2^ (36) = 494.89, *p* < 0.001) accounting for 60.86% of the variance. The two factors were interpreted as Rigid Masculine Role (4 items: 3, 4, 6, 7) and Homophobia/Transphobia (3 items: 2, 5, 9). Along with this, 2 items (1 and 10) showed cross-loading, leading to their exclusion from subscale scoring to maintain factorial standards. Here, H1 (Factor Structure Hypothesis) is strongly supported: the urban Indian sample rewards conformity to traditional masculine norms, which are not unidimensional but have two related yet distinct dimensions that involve public or private role enforcement in institutional domains and perceptions of men’s sexuality. Confirmatory Factor Analysis (CFA) affirmed this two-factor model, revealing excellent fit (*χ*^2^ (13) = 16.29, *p* = 0.234; CFI = 0.991; TLI = 0.985; RMSEA = 0.041). All factor loadings were significant and substantial (Figure 2). The two factors were moderately correlated (*r* = 0.524, *p* < 0.001), indicating they are related concepts but empirically differentiated.

The minimum sample size of 100–150 is recommended for Exploratory Factor Analysis (EFA) when similarity is high and variables per factor are few (Kyriazos, 2018; MacCallum et al., 1999; de Winter et al., 2009; Wolf et al., 2013). Given the two factor structure with 3–4 items per factor and needed high factor loadings (>0.70), a sample of 150 was considered adequate for exploratory analysis. For Conformity Factor Analysis (CFA), while larger sample (N > 200) are preferred (Kline, 2016), this study sample reached its threshold, and all fit indices outdid expected cutoffs (CFI > 0.95, RMSEA < 0.05), proving that the model was well-identified.

CFA for the 10-item Emotion Regulation Questionnaire (ERQ) revealed a borderline fit [*χ*^2^ (34) = 65.092, *p* = 0.001; CFI = 0.908; TLI = 0.879; RMSEA = 0.078]. One item (item 1) has been removed due to poor factor loading (0.356). A revised 9-item model demonstrated acceptable fit [*χ*^2^ (26) = 42.150, *p* = 0.023; CFI = 0.961; TLI = 0.943; RMSEA = 0.062]. The revised 5-item cognitive reappraisal (*α* = 0.735) and the 4-item expressive suppression (*α* = 0.731) are both considered to have acceptable internal consistency.

### 5.2 Phase 2: descriptive and correlational analysis

A Pearson correlation analysis was used to measure bivariate relationships. Table 2 indicates that cognitive reappraisal (CR) and expressive suppression (ES) are strongly positively correlated (*r* = 0.422, *p* < 0.01), and two TMN factors are moderately correlated (*r* = 0.440, *p* < 0.01). Indicating no significant correlation between TMN factors and either ER strategy, rejecting H2. Rigid masculine roles showed a negative correlation with age (*r* = −0.216, *p* < 0.01) and annual income (*r* = −0.178, *p* < 0.01).

**Table 2:** Bivariate relationships among all the variables.

Variables	ES	CR	Age	Marital status	Annual income	Rigid masculine role	Homophobia/Transphobia
ES	1	0.422^**^	−0.045	0.055	−0.015	0.114	0.106
CR	0.422^**^	1	0.111	0.034	0.040	−0.011	0.105
Age	−0.045	0.111	1	−0.610^**^	0.566^**^	−0.216^**^	−0.094
Marital Status	0.055	0.034	−0.610^**^	1	−0.415^**^	0.161^*^	0.088
Annual Income	−0.015	0.040	0.566^**^	−0.415^**^	1	−0.178^*^	−0.134
Rigid masculine role	0.114	−0.011	−0.216^**^	0.161^*^	−0.178^*^	1	0.440^**^
Homophobia/ Transphobia	0.106	0.105	−0.094	0.088	−0.134	0.440^**^	1

*p < 0.05, **p < 0.01, ES, Expressive Suppression; CR, Cognitive Reappraisal.

For the critical TMN–ER correlation, the 95% confidence intervals were Rigid Masculine Role with CR: *r* = −0.011, 95% CI [−0.170, 0.148]; Rigid Masculine Role with ES: *r* = 0.114, 95% CI [−0.045, 0.269]; Homophobia/Transphobia with CR: r = 0.105, 95% CI [.-054, 0.260]; Homophobia/Transphobia with ES: *r* = 0.106, 95% CI [−0.053, 0.261]. All confidence intervals include zero and are relatively narrow (maximum width 0.314), suggesting that any true correlation is likely small in magnitude.

To test H3 (Institutional Embeddedness Hypotheses), two multiple regressions were performed to predict ERQ based on demographics. For CR, *F* (3, 147) = 1.453, *p* = 0.230, and *R*^2^ = 0.029, and for ES, *F* (3, 147) = 0.174, *p* = 0.914, and *R*^2^ = 0.004. The test resulted in no demographic variable being a significant predictor, which clearly rejects the hypothesis H3a. The same test was conducted to examine the relationship between TMN and demographics, revealing a significant association with rigid masculine roles [*F* (5, 145) = 2.844, *p* = 0.018, *R*^2^ = 0.089] and with homophobia/transphobia [*F* (5, 145) = 1.377, *p* = 0.236]. The only significant unique predictor is religious affiliation, with coefficients of (*β* = −0.188, *p* = 0.022) and (*β* = −0.165, *p* = 0.049), indicating that non-religious respondents endorsed fewer traditional masculine norms. This finding partially supports hypothesis H3b, suggesting that key institutional variables like religious beliefs influence masculine ideology.

Supporting this conclusion, chi-square tests revealed a significant association between religious affiliations and masculinity profiles, *χ*^2^ (20) = 31.54, *p* = 0.048, and marital status, *χ*^2^ (10) = 21.20, *p* = 0.020. This indicates that the ‘high on rigid masculine role only’ factor was mostly married men (93%) and exclusively Hindu (100%), and the high homophobic/transphobic only factor was mostly single men (54%) with a higher share of Christians (62%). The higher share of both factors was predominantly among married people (84%) with a mixed religious background. One-way ANOVA results indicate that there are no significant differences in CR or ES scores across marital status [CR: *F* (2, 148) = 0.441, *p* = 0.644; ES: *F* (2, 148) = 0.236, *p* = 0.790] or income groups [CR: *F* (4, 146) = 0.855, *p* = 0.493; ES: *F* (4, 146) = 0.529, *p* = 0.714], reinforcing the findings related to H3a.

### 5.3 Phase 3: analyses of masculine and emotion regulation profiles

Respondents have been classified into four distinct masculinity profiles and emotion regulation profiles based on their scores from the two validated subscales, with the 75th percentile as a cut-point and based on the median splits (CR median = 4.8; ES median = 4.6).

**Figure 3:**
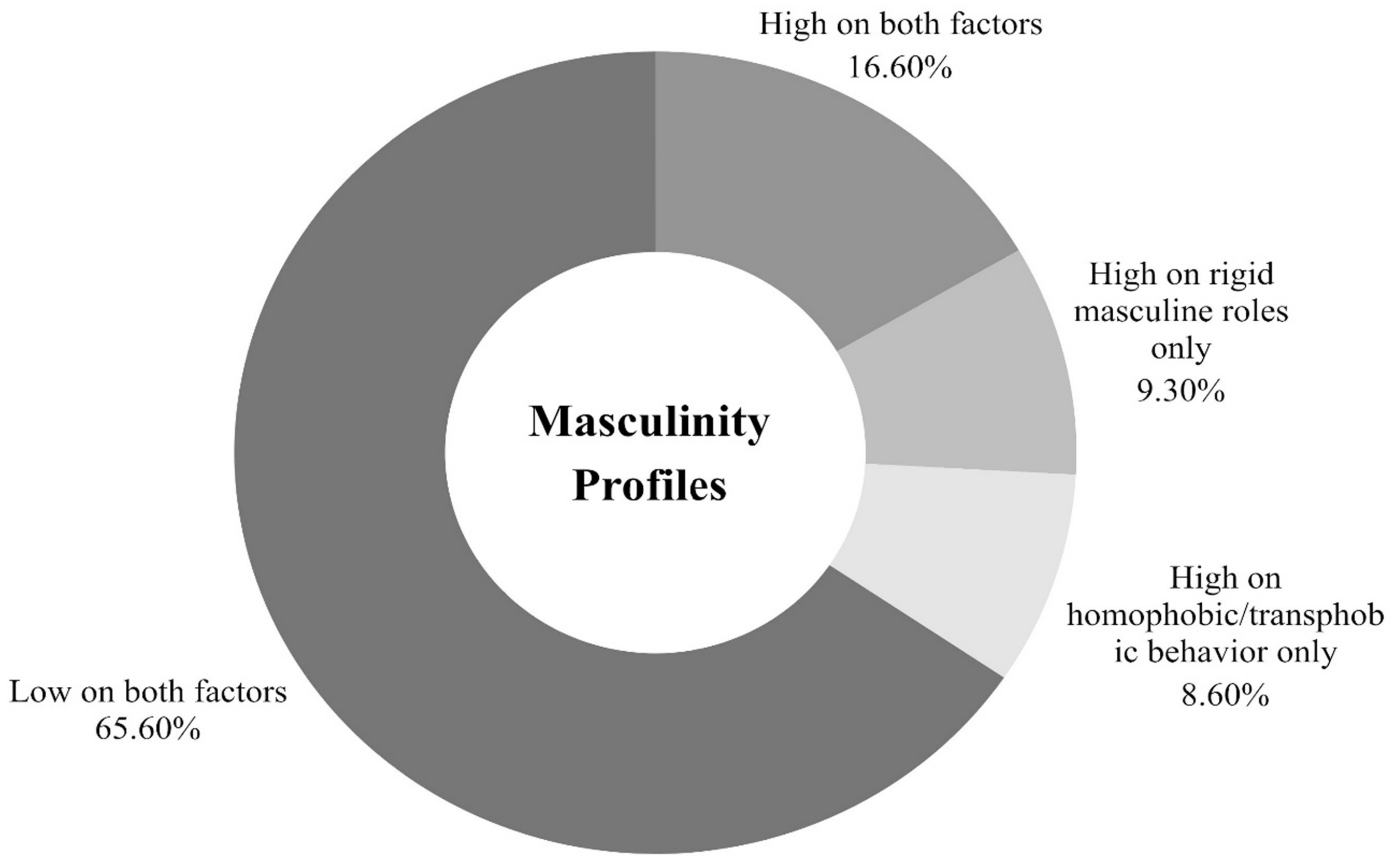
Distribution of masculinity profiles with 75th percentile cut-point.

The above analysis (Figure 3) of the masculinity profile reflects that a significantly high percentage (65.6%) of respondents from this particular sample endorse “Low on Both Factors”. This suggests a potential shift in these norms, indicating that the population is experiencing a predominant change in gender attitudes. The identification of four distinct profiles further complicates simplistic notions of masculinity, indicating that rigid masculine roles and prejudice against homosexuals and transgenders are related but separable constructs.

**Figure 4:**
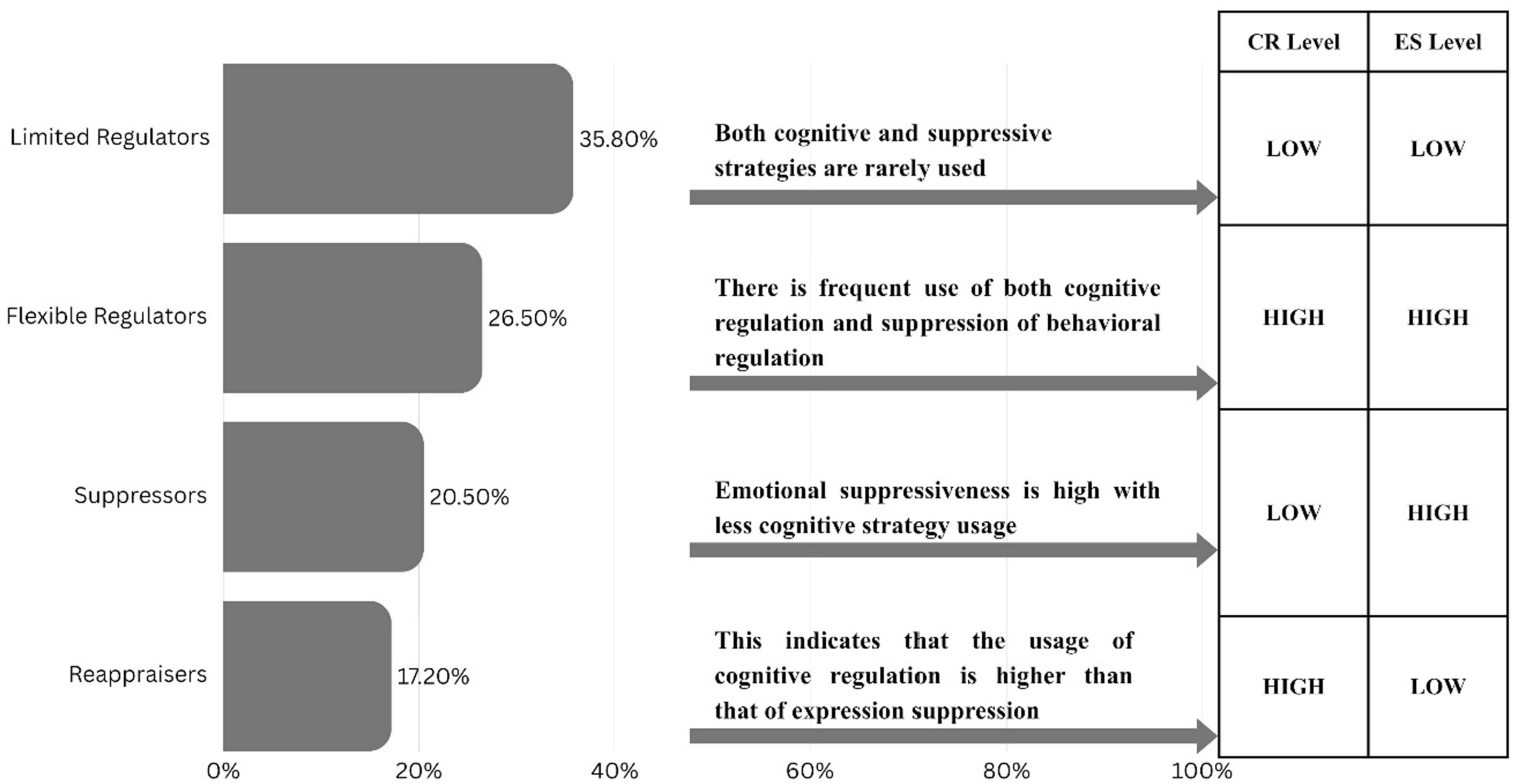
Percentage of distribution of emotion regulation profiles (*N* = 151) using median splits.

The analysis of emotion regulation profiles (Figure 4) indicates that the population can be categorized into two groups: those with limited engagement in regulation strategies (35.8%) and those with flexible engagement using multiple strategies (26.5%), while exclusive use of single strategies is relatively uncommon. The significant correlation between cognitive reappraisal and expressive suppression suggests that these strategies function as complementary rather than compensatory in this sample.

## 6 Discussion

The primary objective of the study was to examine the complex relationships between traditional masculine norms, emotion regulation strategies, and demographic factors among urban Indian men in the city of Chennai. The comprehensive analysis elucidates several key findings that both challenge and confirm the existing theoretical frameworks. First, testing the psychometric validation for both Traditional Masculine Norms (TMN) and the Emotion Regulation Questionnaire (ERQ) reflects an excellent psychometric property in this particular population, with small modifications like the removal of one item from each scale. To measure the core constructs and to adapt with required cultural sensitivity, it is an integral step to remove poorly loaded items in cross-cultural scale adaptation (He and Van de Vijver, 2012; Van de Vijver and Leung, 2021). This enables the scales maintain factorial validity in diverse cultural contexts (Byrne, 2016; Matsumoto and Yoo, 2006). Second, TMN was best categorized by two distinct but related factors: adherence to rigid masculine roles (emotional stoicism, domesticity, encompassing leadership, and anti-feminism) and homophobic/transphobic attitudes. This finding suggests that in the educated urban Indian context, both factors are related but distinct psychological domains. This finding also supports sociological ideas about hegemonic masculinity theory, which suggests that both factors are maintained by men following key norms and through the subordination of marginalized masculinities and femininities (Connell, 2020; Messerschmidt, 2018). Hegemonic masculinity works as a relational construct, defined not only by what it prescribes but also by what it excludes and subordinates (Donaldson, 1993; Hearn, 2004). The moderate correlation (*r* = 0.524) between the factors indicates that the masculinity profile is complex, where they show variance but are not synonymous, such as men who endorse rigid roles but express lower homophobia, or vice versa. Third, one of the most important findings explicates that there is no significant correlation between Traditional Masculine Norms (TMN) and the Emotion Regulation Questionnaire (ERQ). This contradicts from Western studies of a strong correlation between traditional masculinity and emotional suppression. Fourth, the study reflects that demographic factors have a poor correlation with both TMN and ERQ.

There is no significant relationship between TMN and emotion regulation strategies, which contradicts the hypotheses tested in Western literature (Hammond, 2012; Levant and Wimer, 2014; Lynch et al., 2018; McKenzie et al., 2018; Rice et al., 2011; Tager et al., 2010; Wong et al., 2017). This sample of urban Indian men reflects that both TMN and ERQ are independent in their construct. Cultural specificity suggests that cultural norms regarding emotion expression and gender role expectations can influence the psychological effects of gender ideologies. The construction of masculinity among the Chennai urban-based sample may have different emotional models compared to Western models and might be more compatible with emotional expressivity in family and rational contexts (Chen et al., 2025; Dasgupta and Gokulsing, 2014; Tamir et al., 2024), or emotional expression is governed more by context-specific “feeling rules” (Dochania and Dochania, 2025; Hochschild, 1983; Russell Hochschild, 2022) In Indian cultural contexts, emotional expression is administrated by situation and role-based duty (*dharma*) rather than internalized masculine ideology. For instance, a man might suppress grief at a public funeral to sustain family dignity but be allowed to express the same grief and vulnerability in a private ritual setting. This pattern correlate with the “compartmentalization” hypothesis documented across multiple cultural contexts (Dasgupta and Gokulsing, 2014; Israel Meth and Wong, 2023; Van Laar et al., 2026; Nordin et al., 2024; Vierra et al., 2023).

The study, therefore, tests traditional masculine deficit models and recommends a more reflexive psychological framework. Specifically, this methodological approach clearly elucidates that four distinct masculinity patterns in profile analysis may remain incomplete in predicting emotion regulation, meaning masculine ideologies often do not convert to emotional regulation differences. In addition, the four distinct masculinity profiles (Figure 3) did not indicate any significant differences in their use of emotion regulation strategies. This result reflects that even extremely different ways of contemplating masculinity do not always lead to different ways of dealing with emotions. In this specific cultural context, masculine norms exhibit a weak correlation with emotional processes. It suggests that the psychological function of emotion regulation operates with significant autonomy from gendered belief systems, indicating that individuals may manage their emotions effectively regardless of their adherence to traditional masculine norms. In this study, expressive suppression may be a indicator of masculine strain or emotional disfunction but rather a prosocial that demonstrates maturity, respect, and consideration for others (Butler et al., 2003; Soto et al., 2011).

The finding further explicates that 65.6% of respondents scored low in conformity to traditional masculine norms, which encompass both rigid masculine roles and homophobic/transphobic attitudes, thereby challenging stereotypes of uniformly traditional gender attitudes in India. However, these findings cannot be generalized to rural or other regional populations in India without appropriate study. These results suggest that there might be a cultural shift, specifically among the educated urban male population. Four distinct profiles have been derived (Figure 3), and this distribution conveys that there is ongoing change in social dynamics. Several studies indicate that there is a cultural shift in gender attitudes among educated urban Indians (Chopra, 2007; Srivastava, 2015b). Rapid urbanization, educational expansion, and exposure to gender-sensitized knowledge through digital connectivity and media have likely led to more flexible gender attitudes, the embrace of diverse gender norms beyond the traditional patriarchal framework, and positive changes in attitudes toward sexual minority rights among the male population (Ashwani, 2025; Philip, 2022; Bawa, 2025). However, these findings have to be scrutinized with an appropriate cultural lens, as this urban, educated, male population sample is likely to express a shift in attitude toward gender norms.

When testing the multiple regression analyses between demographic factors and TMN and ERQ, the results indicate that none of the individual demographic variables, like age, marital status, annual income, or community (caste), had any significant relationship with ERQ. The ANOVA results further confirmed this pattern. This finding challenges the notion of demographic variables acting as primary drivers of emotion regulation development. Complex psychological experiences, rather than social position, may determine the function of emotion regulation strategies. In contrast, TMN respondents with a religious affiliation were the only group for whom both rigid masculine roles and homophobic/transphobic attitudes were significant predictors. This suggests that non-religious respondents exhibit less conformity to traditional masculine norms. It is also elucidated that religious institutions play a decisive role in the construction of gender ideologies through rituals, community norms, and teachings (Derné, 2005; Osella and Osella, 2006), which can reinforce traditional masculine roles and contribute to the prevalence of homophobic/transphobic attitudes among their adherents. According to the Indian context, religious identity serves as a primary source for gender socialization (Basu, 2015; Chakraborty, 2016).

Chi-square analyses further affirmed this result, reflecting a significant correlation between religious affiliation, masculinity profiles, and marital status. This analysis reflects specific patterns: the “high rigid masculine role only” (9.3%) profile predominantly consists of married men who exclusively practice Hinduism, suggesting that they follow orthodox family rules through hegemonic ownership of property and people, where the resources and social status are owned by male dominance. This hegemony is fundamentally institutionalized within a heterosexual, highly religious family by controlling women’s sexuality, safeguarding male reproductive ownership to maintain lineage purity, which is essential for both caste and community purity, and reproducing the social order that enforces exclusion (Thoty, 2025; Vijayan, 2019). Moreover, Hindu nationalist ideology has an important effect on developing gendered concepts, particularly by reinforcing traditional gender roles and expectations that align with patriarchal values within the community (Banerjee, 2005; Vijayan, 2019). Therefore, the connection between masculinity and religion is not only a cultural aspect, but it is also associated with structures of ownership, violence, and social reproduction. While the “high homophobic/transphobic only” profile mainly includes single men, with three-fourths identifying as Christians, which indicates the influence of particular conservative doctrinal teachings on sexuality within their community, it often promotes a heteronormative view where heterosexual marriage is idealized, encouraging traditional gender roles and heterosexual masculinity. Thus, studies indicate that religious heterosexual men exhibit greater homophobia than heterosexual women (Lakshmi, 2022; Roum et al., 2025). Along with this, marriage in Indian society creates specific gender role expectations that may continuously reinforce the traditional masculine norms, such as the expectation for men to be the primary breadwinners and decision-makers in the household.

Overinterpreting our null findings as definitive evidence against the Gender Role Strain Paradigm. There are chances that the null results may reflect sample characteristics or statistical power limitations rather than a true absence of relationships. Before reaching theoretical conclusions, cross-cultural research requires more cumulative evidence from multiple studies (Van de Vijver and Leung, 2021). Therefore, researchers should view this study as a single data point in the field of masculinity and emotion in non-Western contexts.

### 6.1 Limitations

The study sample of 151 provides 80% power to detect a medium-sized correlation (*r* = 0.22) at *α* = 0.05 (two-tailed). The found TMN & ER correlations ranged from −0.011 to 0.114, all below this threshold. Therefore, we consider the narrow confidence intervals in the correlation estimates as evidence that there is likely a small magnitude in the relationship between TMN and ER in this population. To confirm these preliminary findings, replication with larger samples is needed.

The limited sample size covers only urban, educated populations from South India, which restricts the generalization to rural, non-educated, or other regional populations and other Indian cities without replication. The cross-sectional data cannot capture detailed relationships or developmental trajectories. Moreover, the emotion regulation scale only focuses on general emotional aspects, which does not measure specific emotional expressions, such as anger, sadness, or joy, that may vary significantly across different cultural contexts.

## 7 Conclusion

The study examined two research questions. One, is the Western concept of a link between traditional masculine norms and expressive suppression applicable among urban Indian men? Findings show that there is no significant correlation between TMN and either cognitive reappraisal or expressive suppression, raising questions about the universal applicability of the Gender Role Strain Paradigm in this cultural context.

Two, do demographic factors influence masculine norms and emotion regulation in India? Findings show a partial result: while demographic variables did not predict emotion regulation strategies, religious affiliation significantly predicted TMN conformity. Non-religious individuals have less endorsement toward traditional norms, and distinct masculinity profiles are connected with specific religious identities.

The preliminary findings from this study suggest that masculine norms and emotion regulation functions independently in this urban Indian sample. Emotion regulation may work based on cultural and situational norms rather than as an expression of global masculine identity. The study contributes to the decolonization of sociology and psychological sciences, once widely believed to be culturally specific. Thus, future research on men’s studies in India could focus on identifying factors that are linked with masculine ideologies, roles, and men’s health and emotion through intersectional research approaches. The study, therefore, encourages researchers to conduct more extensive cross-cultural, multi-site studies and in-depth research on masculinity in India, with the aim of developing Indian versions of these scales.

## Data Availability

The raw data supporting the conclusions of this article will be made available by the authors, without undue reservation.
